# In silico design and analyses of a multi-epitope vaccine against Crimean-Congo hemorrhagic fever virus through reverse vaccinology and immunoinformatics approaches

**DOI:** 10.1038/s41598-022-12651-1

**Published:** 2022-05-24

**Authors:** Akinyemi Ademola Omoniyi, Samuel Sunday Adebisi, Sunday Abraham Musa, James Oliver Nzalak, Zainab Mahmood Bauchi, Kerkebe William Bako, Oluwasegun Davis Olatomide, Richard Zachariah, Jens Randel Nyengaard

**Affiliations:** 1 Department of Human Anatomy, Faculty of Basic Medical Science, College of Medical Sciences, Ahmadu Bello University, Zaria, Nigeria; 2 Department of Veterinary Anatomy, Faculty of Veterinary Medicine, Ahmadu Bello University, Zaria, Nigeria; 3 Department of Human Anatomy, Faculty of Basic Medical Sciences, Abubakar Tafawa Balewa University, Bauchi, Nigeria; 4 Department of Clinical Medicine, Core Centre for Molecular Morphology, Section for Stereology and Microscopy, Aarhus University, Aarhus, Denmark; 5 Department of Pathology, Aarhus University Hospital, Aarhus, Denmark

**Keywords:** Protein design, Computational biology and bioinformatics

## Abstract

Crimean Congo Hemorrhagic Fever virus (CCHFV) is a deadly human pathogen that causes an emerging zoonotic disease with a broad geographic spread, especially in Africa, Asia, and Europe, and the second most common viral hemorrhagic fever and widely transmitted tick-borne viral disease. Following infection, the patients are presented with a variety of clinical manifestations and a fatality rate of 40%. Despite the high fatality rate, there are unmet clinical interventions, as no antiviral drugs or vaccines for CCHF have been approved. Immunoinformatics pipeline and reverse vaccinology were used in this study to design a multi-epitope vaccine that may elicit a protective humoral and cellular immune response against Crimean-Congo hemorrhagic fever virus infection. Three essential virulent and antigenic proteins (S, M, and L) were used to predict seven CTL and 18 HTL epitopes that were non-allergenic, antigenic, IFN-γ inducing, and non-toxic. The epitopes were connected using linkers and 50S ribosomal protein L7/L12 was used as an adjuvant and raised a multi-epitope vaccine (MEV) that is 567 amino acids long. Molecular docking and simulation of the predicted 3D structure of the MEV with the toll-like (TLR2, TLR3, and TLR4) receptors and major histocompatibility complex (MCH-I and MCH-II) indicate high interactions and stability of the complexes, MM-GBSA free binding energy calculation revealed a favourable protein–protein complex. Maximum MEV expression was achieved with a CAI value of 0.98 through in silico cloning in the *Drosophila melanogaster* host. According to the immune simulation, IgG1, T-helper cells, T-cytotoxic cells, INF-γ, and IL-2 were predicted to be significantly elevated. These robust computational analyses demonstrated that the proposed MEV is effective in preventing CCHFV infections. However, it is still necessary to conduct both in vitro and in vivo experiments to validate the potential of the vaccine.

## Introduction

After dengue fever, Crimean-Congo Hemorrhagic Fever (CCHF) is the second most common viral hemorrhagic fever and the world's most widely transmitted tick-borne viral disease^1^. It's characterised by high fever, headache, weakness, nausea, vomiting, and diarrhoea, as well as elevated liver enzymes, elevated levels of creatine phosphokinase (CPK), and lactate dehydrogenase (LDH), and disturbed haemostasis^2^. Despite presentation as a low fever in the majority of cases, some patients develop severe hemorrhagic disease^3^. CCHF was first described in humans as a disease in the 1940s when soldiers and farmers in the Crimean Peninsula became ill with a hemorrhagic disease^4^.

CCHF is an emerging zoonotic disease with a broad geographic spread (much of Africa, Asia, and Europe)^5^ and a 40% fatality rate^6^. It is caused by the Crimean Congo Hemorrhagic Fever virus (CCHFV), a negative-sense RNA arbovirus (Arthropod-borne virus)^7^ belonging to the genus Nairovirus and family Bunyaviridae, which is transmitted by ticks^8^. Humans can become infected with CCHFV by tick bites, crushing infected ticks, via inhalation, or through unprotected contact with body fluids of infected animals or humans^9^.

In the late 1960s, the Crimean-Congo hemorrhagic fever virus was named after the discovery that the causative agent of hemorrhagic disease in Zaire (the current Democratic Republic of Congo) was similar to that of the hemorrhagic disease in the Crimea^10^. Based on phylogenetic analysis of the complete genetic sequence of the S RNA segment of the genome and geographical origin, up to nine genetically distinct clades have been reported, suggesting a high genetic diversity^11,12^. Evidence suggests that the viral gene segments are often reassorted, possibly as a result of animal trade between African and Asian regions^13^.

In a variety of mammalian hosts, the virus induces a temporary viremia^14^. In comparison to humans, immune-competent mammals, do not show symptoms of disease^15,16^. Animal models and treatment trials against CCHF have been delayed as a result of this. Retinoic acid-inducible gene I (RIG-I)^17^, Toll-like receptors (TLRs)^18^, and nuclear factor-kappa B^19^ may act as innate immune sensors of CCHFV. Reduction in the replication of CCHFV by treatment with interferon in interferon-signalling competent cells and the ability of CCHFV to cause severe disease in mice deficient in the type I interferon system but not wild-type (WT) mice suggests that CCHFV is an interferon sensitive virus^20,21^. This implies that host innate immune responses in vertebrate species play a substantial role in limiting CCHFV pathogenesis^22,23^.

The viral genome consists of three RNA segments: small (S) encoding the viral nucleoprotein (NP), medium (M) encoding the glycoprotein precursor (GPC) that yields the structural glycoproteins (GN and GC), and large (L) encoding the RNA-dependent RNA polymerase^24^. Following interaction between its glycoprotein (GN and GC) and the host cell's receptor, CCHFV releases its genome after entering the cell through endocytosis. Once within the cell, genomic fragments are uncoated and transcribed into viral mRNA by the L protein, which is then converted into NP and L proteins used for genomic RNA replication, resulting in genomic ribonucleoprotein complexes (RNP)^25^. Within the endoplasmic reticulum (ER) and Golgi bodies, newly synthesized CCHFV particles are processed and matured before being released via exocytosis^26^.

Immunoinformatics in vaccine development provides a quick, reliable, and efficient approach to disease vaccine development^27^. The antigenicity of pathogen secretory proteins makes them an excellent candidate for predicting B and T cell epitopes in vaccine production^28,29^. Despite the high mortality rate, no antiviral drugs or vaccines for CCHF have been approved^30,31^. Although efforts have been made in vaccine development with the Bulgarian vaccine, DNA vaccine and viral nucleoprotein, the extensibility and safety issues with this type of vaccine have likely prevented widespread use, necessitating the development of new vaccine platforms for CCHFV^8,32^. This study aims to use in silico immunoinformatics pipeline and reverse vaccinology to design a multi-epitope vaccine that may elicit a protective humoral and cellular immune response against Crimean-Congo hemorrhagic fever virus infection.

## Methodology

**Figure 1:**
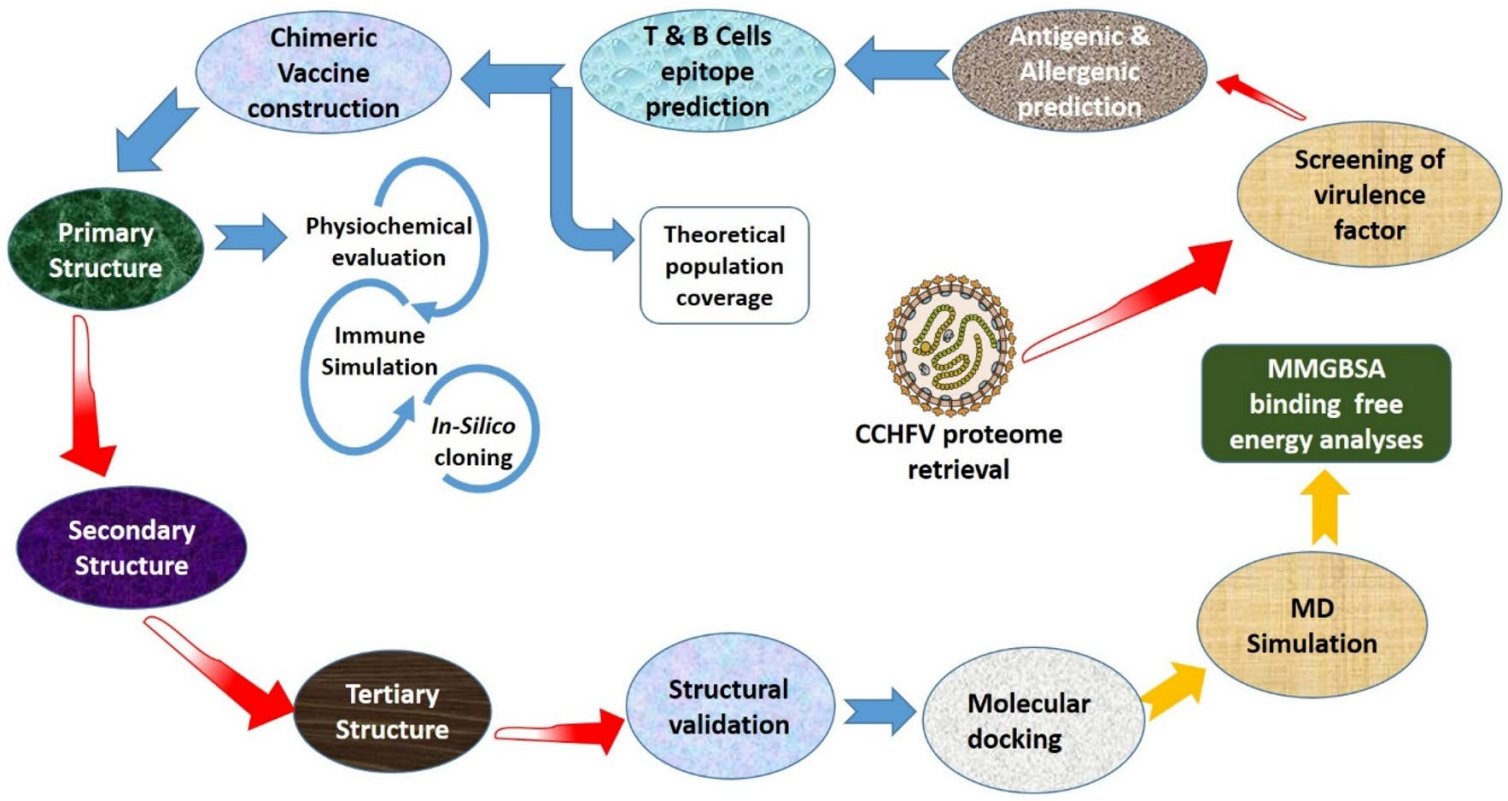
The overall flow of the work was created using Microsoft Office (PowerPoint) Professional Plus 2019. The methodology was divided into eight parts: CCHFV proteome retrieval, screening of virulence factor, prediction of epitopes (CTL and HTL), multi-epitope vaccine construction, 3D structure modelling, molecular docking and dynamic simulation, in-silico expression, and immune stimulation.

The reverse vaccinology and immunoinformatics pipeline used included eight major components: CCHFV proteome retrieval, virulence factor screening, epitope prediction (CTL and HTL), multi-epitope vaccine design, 3D structure modelling, molecular docking and dynamic simulation, in-silico expression, and immune simulation (Fig. 1).

### Retrieval of CCHFV proteome

The complete amino acid sequence of the CCHFV large segment (L) (NC_005301.3)^33^, glycoprotein precursor (GPC) (NC_005300.2)^34^, and nucleoprotein (NP) (NC_005302.1) were retrieved from Gene Bank Database, NCBI (https://www.ncbi.nlm.nih.gov/) in standard FASTA format.

### Screening of virulence factor

Based on manually annotated training data consisting of bacterial and viral protective antigens and an optimised supervised machine learning model, Vaxign-ML predicts the protegenicity score that is validated through nested five-fold cross-validation. For the L, GPC, and NP of CCHFV, Vaxign-ML (http://www.violinet.org/vaxign2) was used to compute the protective antigenicity (protegenicity) score and predict subcellular localization, transmembrane helix, and adhesion probability^35^.

### Prediction of cytotoxic T lymphocytes (CTL) epitope

CTL epitopes for L, GPC, and NP of CCHFV were predicted at a threshold of 0.75 using an online server NetCTL 1.2 (http://www.cbs.dtu.dk/services/NetCTL/). It predicts CTL epitopes based on MHC-I binding peptide prediction, proteasomal C-terminal degradation activity using an artificial neural network, and Transporter Associated with Antigen Processing (TAP) employing weight matrix^36^. Predicted CTL epitopes were subjected to the Class I immunogenicity server (http://tools.iedb.org/immunogenicity/) using the default setting to select the best epitopes for 9-mer peptides for class I immunogenicity^37^.

### Prediction of helper T-cell epitopes

HTL epitopes for seven human alleles (HLA-DRB1*03:01, HLA-DRB1*07:01, HLA DRB1*015:01, HLA-DRB3*01:01, HLA-DRB3*02:02, HLA-DRB4*01:01, and HLA-DRB5*01:01) were predicted for L, GPC, and NP of CCHFV by MHC-II prediction module of the online server IEDB (http://tools.iedb.org/mhcii/)^38,39^ based on receptor affinity derived from the IC50 value (binding score) given to each epitope. Where IC50 value < 50 nM denotes high binding affinity, IC50 score < 500 nM denotes moderate and IC50 value < 5000 nM signifies low binding affinity of predicted epitopes. The score of binding affinity of the epitope is inversely proportional to the percentile rank, which means that the higher the binding affinity, the lower the percentile rank. Predicted HTL epitopes were further subjected to the IFN epitope server (http://crdd.osdd.net/raghava/ifnepitope/index.php)^40^ using the SVM hybrid and Motif as the approach and IFN-gamma against other cytokines as the model of prediction. This was done to validate the ability of the predicted HTL epitopes to cause IFN-γ production which has been reported to minimize host damage, protect against infectious diseases and inhibit viral replication after activation of helper T cells^41–43^.

### Allergenicity prediction

AlgPred (http://www.imtech.res.in/raghava/algpred/)^44^ an online server that uses six different approaches for the prediction of allergenicity was used to predict the allergenic score for the predicted CTL epitopes, HTL epitopes and the vaccine construct with high accuracy of 85% at a 0.4 threshold.

### Antigenicity prediction

The antigenicity of the predicted CTL epitopes, HTL epitopes, and vaccine construct was done with high accuracy based on the physicochemical properties of a given amino acid sequence using the VaxiJen online server (http://www.ddg-pharmfac.net/vaxijen/VaxiJen/VaxiJen.html)^45^ at a threshold of 0.4 (for virus selected as target organism).

### Toxicity prediction

ToxinPred an online server (http://crdd.osdd.net/raghava/toxinpred/)^46^ that predicts the toxicity of epitopes based on the physicochemical properties was used to screen for non-toxic epitopes.

### Population coverage and alignment analysis

The frequencies of different Human Leucocyte Antigens (HLA) alleles vary with ethnicities^47^, and CCHFV is widely spread in Africa, Asia, the Middle East, and Eastern Europe. The HLA-alleles distribution among the endemic population is essential for effective multi-epitope vaccine development. In this study, the IEDB (http://tools.iedb.org/population/) population coverage analysis tool^48^ was used for population coverage of the potential CTL and HTL epitopes and their MHC binding alleles. The Protein Basic Local Alignment Search Tool (BLASTp)^49^ available at https://blast.ncbi.nlm.nih.gov/Blast.cgi? was used to evaluate the sequence similarity of the query sequences to other sequences available in the National Center for Biotechnology Information (NCBI) database. Multiple alignments of the sequences was performed using Clustal Omega available at EMBL-EBI web interface (https://ebi.ac.uk/Tools/msa/clustalo/)^50,51^ and the results were analysed using Jalview version 2.11^52^.

### Multi-epitopes vaccine sequence construction

Based on the result of the aforementioned procedures, the sequence of the vaccine construct was derived from the predicted sequences of the CTL, HTL, and linear B cells epitopes. AAY, GPGPG, and KK linkers were used to join the CTL, HTL, and B cells epitopes respectively^53^. EAAAK linker was used to join the adjuvant to the vaccine construct^54^ using Notepad++ version 8.1.

### B cell epitope prediction

B cell lymphocytes produce antibody molecules that are inserted into the plasma membrane as part of B-cell receptors^55^. B-cell epitopes play a large role in host antibody production by binding to the receptors on B cells. B-cell epitopes play a large role in host antibody production by binding to the receptors on B cells. BepiPred-2.0 (http://tools.iedb.org/bcell/)^56^ was used to predict these epitopes on an online server that employs a random forest algorithm method trained on epitopes annotated from antibody-antigen protein structures. To further predict the conformational B-cell epitopes, DiscoTope 2.0 (http://tools.iedb.org/discotope/)^57^ was used. This is an online server that uses 3D structures to predict discontinuous epitopes based on amino acid statistics, spatial information, and surface accessibility in an accumulated data set of conformational epitopes determined by X-ray crystallography of antibody/antigen protein complexes, as well as contact distances into its potential B-cell epitope prediction along the length of a protein sequence.

### Physiochemical parameters and identification of domain

Physiochemical parameters (theoretical PI, the composition of amino acid, in vitro and in vivo half-life, molecular weight, instability index, aliphatic index and grand average of hydropathicity GRAVY) and solubility of the vaccine construct were predicted using an online server ProtParam (http://web.expasy.org/protparam/)^58^ and the SOLpro tool in the SCRATCH suite (http://scratch.proteomics.ics.uci.edu/)^59^.

### Prediction of secondary structure

PSIPRED (http://bioinf.cs.ucl.ac.uk/psipred/)^60^ was used for high accuracy prediction of the secondary structure of the amino acid sequence in the vaccine construct. It is an online tool that uses position-specific prediction Psi-BLAST to identify and select sequences showing significant homology to the vaccine protein.

### Tertiary structure prediction

Robetta (http://robetta.bakerlab.org) an automated tool that predicts 3D structure models of protein after parsing the structure into respective domains based on either comparative modelling or de novo structure was used for the prediction of the tertiary structure of the construct. For comparative modelling homologs, sequences were used as templates after identification by BLAST, 3D-Jury or FFAS03, and PSI-BLAST. De novo structures were generated using the Rosetta fragment insertion method if homologs were not found^61^.

### Tertiary structure refinement

The tertiary structure for the predicted multi-epitope subunit vaccine construct was refined using an online web tool Galaxy Refine (http://galaxy.seoklab.org/)^62^ that improves protein structure using the CASP10 method for repacking, protein's side chain reconstruction, as well as MD simulations for relaxation of the global and the local quality of the tertiary structure.

### Validation of tertiary structure of vaccine construct

The validation of the tertiary structure of the vaccine construct was done on ProSA-web, SAVES v6.0, and PROCHECK.

Firstly, ProSA-web (https://prosa.services.came.sbg.ac.at/prosa.php) was used to compute the quality score for a specific input structure and displayed it in the context of all known protein structures^63^. The 3D molecule viewer in ProSA-web results facilitates the detection of the problematic part that is, scores lying outside a range characteristic for native proteins. Secondly, another validation server ERRAT in the SAVES v6.0 server (https://saves.mbi.ucla.edu/) was used to generate the overall quality score of the modelled protein by analyzing non-bonded interactions in comparison to reliable high-resolution crystallography structures^64^. Thirdly, analysis and generation of a Ramachandran plot for visualization of allowed and disallowed dihedral angles psi (ψ) and phi (φ) of amino acid-based on the van der Waal radius of the side chains was done using PROCHECK (https://saves.mbi.ucla.edu/)^65^.

### Molecular docking of the vaccine construct with immune receptors

Interaction of an antigen and a specific receptor is required for the initiation of an appropriate immune response. To evaluate the interaction between the vaccine construct and its receptors, a molecular docking approach was used. A 3D structure of MHC I (PDB ID: 6P2F), MHC II (PDB ID: 1AQD), TLR2 (PDB ID: 2Z7X), TLR3 (PDB ID: 4G8A), and TLR4 (PDB ID: 1ZIW) was retrieved from a protein data bank and used as the receptors. The HDOCK server (http://hdock.phys.hust.edu.cn/)^66^ was used to dock the vaccine construct with the receptors. It functions by sampling and calculating the atomic shape portrayal; coordinating surface fixes as well as separating the surface of the putative binding modes between the two proteins using the Fast Fourier Transform-based global search approach^67^ and appraising the sampled binding modes with an enhanced iterative template-based scoring function for protein–protein interaction^68^. To accurately predict the binding strength of the complexes, the PROtein BinDIng enerGY (PRODIGY) server (https://bianca.science.uu.nl/prodigy/)^69^ was utilised to assess binding affinity (ΔG) and the dissociation constant (Kd) in room temperature of the complexes. To visualize the interactions between docked complexes, we used the PDBePisa (https://www.ebi.ac.uk/msd-srv/prot_int/cgi-bin/piserver)^70^ server and Pymol version 2.3^71^.

### Molecular dynamics and free binding energy calculation

Molecular dynamics (MD) simulation was done to minimize and evaluate the stability of the 3D structure of the vaccine construct for probing the stability of the protein–protein complex of the vaccine constructs and the MHC I, MHC II, TLR2, TLR3, and TLR4 was done using Assistant Model Building with Energy Refinement (AMBER 20)^72^. The recommended protein ff19SB force field^73^ with OPC water model^74,75^ was used for the simulation. Octahedron box shape was utilised with the vaccine or resulting complex at least a distance of 12 Å away from the edge of the water-filled box to achieve at least three-layer of solvation on all sides of the protein surface^76^. Na^+^ and Cl^−^ counter ions were applied where necessary to neutralize the system using the “tleap” package of Amber. The systems were minimised at 500 cycles of steepest descent and 1000 steps of a conjugate gradient to remove all constraints atoms. The systems were heated for a period of 50 ps to maintain a constant temperature of 300 K using Langevin dynamics and equilibrated for 5 ns at temperature and pressure with isotropic position scaling to achieve conformational stability. All simulation production was carried out for a period of 100 ns in PMEMD.cuda^77,78^ with the SHAKE and Particle-Mesh Ewald (PME) method, and a non-bond contacts cut-off radius of 10 Å was used for long-term interactions.

Visual Molecular Dynamics^79^ (VMD) and MMPBSA.py^80^ implemented in Amber20 were used for the post-simulation trajectories analysis to evaluate the Root Mean Square Deviation (RMSD)-Eq. (1) and Molecular Mechanics Generalised Born Surface Area(MM-GBSA)-Eq. (2) binding free energy of the complexes.

1 $$ RMSD = \frac{{\sqrt {\sum\nolimits_{i = 0}^{N} {[m_{i} \times (X_{i} - Y_{i} )^{2} ]} } }}{M} $$

where N = number of atoms, m_i_ = mass of atom *i*, X_i_ = coordinate vector for target atom *i*, Y_i_ = coordinate vector for reference atom *i*, and M = total mass. If the RMSD is not mass-weight, all m_i_ = 1 and M = N.

2 $$ {\text{Binding free energy }}\,\left( {\Delta {\text{G}}_{{{\text{bind}}}} } \right) \, = \, \Delta {\text{H }} - {\text{T}}\Delta {\text{S}} $$

where ΔH is the enthalpy change as computed as the sum of changes of the gas-phase energy (ΔE_MM_) and solvation free energy (ΔG_sol_) mean over a conformational ensemble generated by MD simulations. TΔS is the entropic contribution.

### Optimization and in-silico cloning of vaccine construct

For the multi-epitope vaccine construct expression in a selected expression vector, reverse translation and optimization of codons, were conducted in the Java Codon Adaptation Tool (JCat) server (http://www.jcat.de)^81^. To ensure expression of the final vaccine structure in host *Drosophila melanogaster*^82^, codon optimization was performed because the codon usage by *Drosophila melanogaster* is different from that of the native host. The output of JCat consists of a codon adaptation index (CAI) which gives information on codon usage biases. An ideal CAI score is 1.0 but > 0.8 is considered a good score^83^ and the percentage of GC content, ranges between 30–70%. GC content values outside this range suggest unfavourable effects on translation and transcription^84^, which can be used to ascertain the level of protein expression.

### Immune simulation

In silico immune simulations were conducted using the C-ImmSim server (http://150.146.2.1/C-IMMSIM/index.php) to further validate the immunogenic and immune response profile of the vaccine construct^85^. C-ImmSim simultaneously simulates three compartments that represent three separate localization of immune cells in mammals: the bone marrow, the thymus, and a tertiary lymphatic organ, such as a lymph node. It does this by using a position-specific scoring matrix (PSSM) for immune epitope prediction and machine learning techniques for the prediction of immune interactions. At intervals of four weeks^29,86^, three injections were given and all simulation parameters were set at default with time steps set at 1, 84, and 168, where each time step is 8 h and time step 1 is injection at time = 0. To probe for clonal selection, additional 12 injections of the designed vaccine construct were given four weeks apart to mimic repeated antigen exposure seen in a typical endemic area.

### Research involving human participants and/or animals

This article does not contain any studies involved with human participants or animals performed by any of the authors.

## Results

### Retrieval of protein sequences

The viral genome of CCHFV which consists of RNA segments encoding the nucleoprotein, glycoprotein precursor, and RNA-dependent RNA polymerase proteins was retrieved from the GenBank and used for the prediction of CTL and HTL epitopes for the multi-epitope subunit vaccine design. Fifty S ribosomal protein L7/L12 was retrieved from the UniProt database (P0A7K2)^87,88^ and used as an adjuvant for the immune interaction based on its ability to induce antiviral immune response^89^.

### Screening of virulence factor

The encoded segment for NP, GPC, and L proteins of CCHFV was predicted by Vaxign sever as protective antigens with a high protegenicity score. All were predicted to be cytoplasmic proteins and only NP was predicted to have a high adhesive probability among the three proteins (Table 1). Adhesin plays a vital role in the virus adhering to the host cell and enabling the virus entry to the host cell^90^. All the predicted proteins were not similar to human, mouse, or pig proteins.

**Table 1:** Protegenicity score and adhesion probability score of RNA segments of CCHFV.

RNA segments	Protein name	Protegenicity score	Adhesion probability
S	Nucleoprotein	91.2	0.435
M	Glycoprotein precursor	89.6	0.195
L	RNA polymerase	89.6	0.019

### Cytotoxic T lymphocyte (CTL) epitope prediction

The CTL epitopes (9-mer) prediction using the NetCTL v2.0 server yielded 146 epitopes out of which 54 epitopes were predicted to be antigenic, immunogenic, and non-toxic. From these, seven non-overlapping (GPC = 6 and NP = 1) epitopes of human MCH-I alleles HLA-B*35:01, HLA-B*30:02, HLA-A*01:01, HLA-A*02:06, and HLA-B*57:01 were selected based on high immunogenicity scores as CTL epitopes for vaccine construction (Table 2).

**Table 2:** Selected cytotoxic T-lymphocyte (CTL) epitopes for multi-epitope vaccine construction.

ID	Peptide seq	Combine score	Antigenicity	MCH I score	Toxicity
1	QSAQIDTAF	1.005	0.588	0.102	Non-toxic
2	FLFWFSFGY	1.080	1.100	0.338	Non-toxic
3	LKDDEETGY	0.982	0.667	0.274	Non-toxic
4	STANIALSW	0.929	1.314	0.067	Non-toxic
5	GLDCDDTFF	0.915	0.533	0.097	Non-toxic
6	YTSICLFVL	0.816	0.615	0.140	Non-toxic
7	TTMAFLFWF	0.756	0.608	0.314	Non-toxic

### Helper T lymphocyte (HTL) epitope prediction

The HTL epitopes (15-mer) prediction produced 126 non-allergenic epitopes from the CCHFV proteome, out of which 26 were further predicted to be antigenic, non-toxic, and IFN-γ positive. From these, 18 (GPC = 11, NP = 5, and L = 2) non-overlapping epitopes for human alleles HLA-DRB1*03:01, HLA-DRB1*07:01, HLA-DRB1*15:01, HLA-DRB3*01:01, HLA-DRB3*02:02, HLA-DRB4*01:01 and HLA-DRB5*01:01 were considered for vaccine construction based on high percentile rank scores, non-allergenic, antigenic, and non-toxic as HTL epitopes (Table 3).

**Table 3:** Selected helper T lymphocyte (CTL) epitopes for multi-epitope vaccine construction.

S. no	Allele	Peptide	Allergenicity	Antigenicity	Toxicity	IFN-γ
1	HLA-DRB5*01:01	FRATMEVSNRALFIR	− 0.419	0.438	Non-toxic	Positive
2	HLA-DRB5*01:01	CKLMCFRATMEVSNR	− 0.449	1.150	Non-toxic	Positive
3	HLA-DRB3*02:02	FYLLIIVGTLGKRLK	− 0.426	1.205	Non-toxic	Positive
4	HLA-DRB1*07:01	APIGQGKTIEAYRAR	− 0.502	0.698	Non-toxic	Positive
5	HLA-DRB3*02:02	FLFWFSFGYVITCIL	− 0.424	0.721	Non-toxic	Positive
6	HLA-DRB5*01:01	AIFYLLIIVGTLGKR	− 0.523	1.084	Non-toxic	Positive
7	HLA-DRB3*02:02	EHPESLTQSATPGLM	− 0.543	0.476	Non-toxic	Positive
8	HLA-DRB5*01:01	ELGCYTINRVRSFKL	− 0.607	0.950	Non-toxic	Positive
9	HLA-DRB4*01:01	ESTGVALKRSSWLIV	− 1.149	1.431	Non-toxic	Positive
10	HLA-DRB1*03:01	GLQLINITRHSTRIV	− 0.468	0.853	Non-toxic	Positive
11	HLA-DRB1*03:01	GVALKRSSWLIVLLV	− 0.487	1.362	Non-toxic	Positive
12	HLA-DRB3*01:01	GRSGIALVATGLAKL	− 0.512	0.850	Non-toxic	Positive
13	HLA-DRB3*01:01	MHPAVLTAGRISEMG	− 1.438	1.133	Non-toxic	Positive
14	HLA-DRB3*01:01	RIYMHPAVLTAGRIS	− 1.030	0.785	Non-toxic	Positive
15	HLA-DRB1*03:01	SFQQNRIYMHPAVLT	− 0.598	0.546	Non-toxic	Positive
16	HLA-DRB1*07:01	NKSGRSGIALVATGL	− 0.762	1.008	Non-toxic	Positive
17	HLA-DRB1*15:01	AVEDLILMLTGRAVK	− 0.751	0.624	Non-toxic	Positive
18	HLA-DRB1*15:01	DLILMLTGRAVKPSA	− 0.233	0.815	Non-toxic	Positive

*IFN-γ* interferon-gamma.

### Allergenicity and antigenicity prediction of the multi-epitope vaccine construct

The allergenicity of the vaccine construct was predicted by using the AlgPred server, which found the multi-epitope vaccine construct to be non-allergenic (Allergenicity score − 1.028). While an antigenic score of 0.593 was derived as predicted by the VaxiJen server. These results suggest that our multi-epitope vaccine candidate possesses strong antigenic and non-allergenic properties that will provoke the immune response.

### Theoretical population coverage and alignment analysis

**Figure 2:**
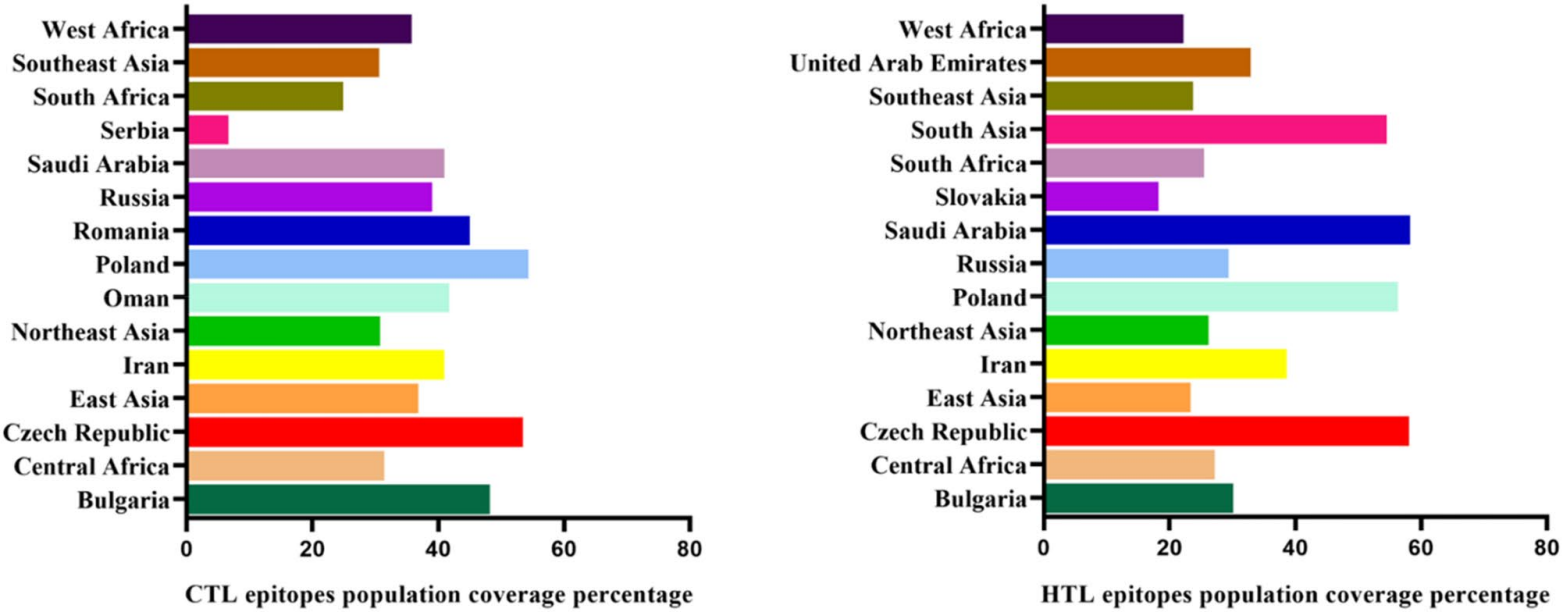
Theoretical population coverage of selected CTL and HTL epitopes allele distribution.

The HLA allele distribution varies between different geographical and ethnic regions around the globe. Hence, population coverage analysis of the selected CTL and HTL epitopes with their corresponding HLA alleles used in the construction of the multi-epitope subunit was considered. The selected HTL and CTL epitopes had widespread coverage of the endemic population of CCHF. The HTL epitopes cover 58.1%, 58.3%, and 56.3% of the Czech Republic, Saudi Arabia, and Poland population, while the CTL epitopes cover 53.6% and 54.3% of the Czech Republic and Poland population, respectively (Fig. 2).

The BLASTp returned a total of 100 sequences for each of the query sequences [nucleoprotein (NP) (NC_005302.1), glycoprotein precursor (GPC) (NC_005300.2), large segment (L) (NC_005301.3)] out of which 2 sequences for not being related to CCHFV. Each of the sequences has an average of 99% sequence coverage and the nucleoprotein has an average of 89.4% identical sequences, glycoprotein was 89.6 and RNA polymerase sequence was 91.8%. 83.3% of the CTL epitopes selected are conserved and HTL epitopes were 62.5% conserved.

### CCHFV multi-epitope chimeric vaccine construction

The construction of the CCHFV chimeric vaccine was done in Notepad++ version 8.1 text editor, the adjuvant protein (50S ribosomal protein L7/L12) retrieved with UniProt ID: P0A7K2 was joined with the first CTL epitope through EAAAK linker. With adjuvant connected to the first CTL epitope, eight CTL and eighteen HTL epitopes were joined using AAY and GPGPG linkers. The respective linkers were introduced to generate sequences with minimised junctional immunogenicity, a high level of expression, and improved bioactivity of the fusion protein (Supplementary Fig. 1). The CCHFV chimeric vaccine is 567 amino acid sequences long.

### B-cell prediction

The BepiPred server predicted twenty linear B-cell epitopes of varying lengths ranging from 1 to 28 amino acid sequences long and the DiscoTope server yielded six discontinuous B-cell epitopes of varying residue length with propensity and Discotope scores ranging from − 4.07 to 1.96 and − 3.66 to 1.72, respectively (Supplementary Fig. 2).

### Prediction of physiochemical parameters

The result from the ProtParam server showed that the multi-epitopes vaccine has a molecular weight of 58.3 kDa and a theoretical protrusion index (PI) of 9.09, which shows the vaccine construct is basic. The estimated in vitro half-life in human mammalian reticulocytes was 30 h and the instability index was computed to be 32.1 revealing that the vaccine construct is a stable protein. The aliphatic index was calculated to be 90.1 indicating a thermostable nature at different temperatures, and the grand average of hydropathicity (GRAVY) was 0.232, thus, indicating that the vaccine construct is hydrophobic (Table 4).

**Table 4:** Physiochemical properties of the subunit multi-epitopes vaccine construct.

S. no.	Physiochemical properties	Results
1	Number of amino acids	567
2	Molecular weight	58.3 kDa
3	Theoretical protrusion index (PI)	9.09
4	Estimated half-life (mammalian reticulocytes, in vitro)	30 h
5	Estimated half-life (yeast, in vivo)	> 20 h
6	Estimated half-life (*Escherichia coli*, in vivo)	> 10 h
7	Instability index	32.1
8	Aliphatic index	90.1
9	Grand average of hydropathicity (GRAVY)	0.232
10	Solubility upon overexpression	0.982

### Prediction of secondary structure

**Figure 3:**
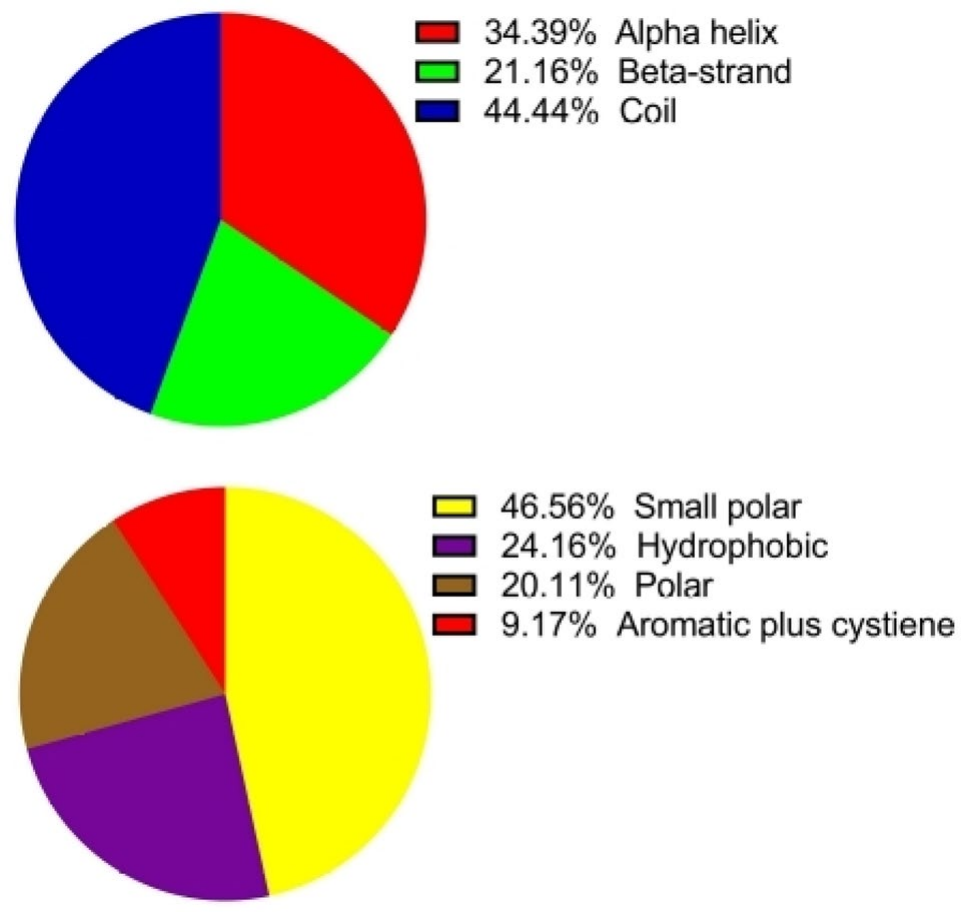
Graphical representation of the secondary structure of the subunit multi-epitopes vaccine construct predicted by the PSIPRED server.

The secondary structure of the vaccine construct as predicted by the PSIPRED server shows that the multi-epitopes vaccine is composed of a high percentage of the coil (44.4%) when compared with alpha-helix (34.4%) and beta-strand (21.2%). The vaccine construct also contains small polar residues (24.2%), hydrophobic (24.2%), polar (20.1%), and aromatic plus cysteine (9.2%) (Fig. 3).

### 3D structure modelling, refinement, and validation

Based on a multi-templates approach, the RaptorX server-generated five 3D structure models of the multi-epitopes construct with RMSD ranging from 11.1 to 15.5 Å. The best model was selected based on its RMSD for further analysis.

Using the GalaxyRefine server for the refinement of the 3D structure of the chimeric vaccine, five models were produced. Model 1 was selected based on its model quality score including GDT-HA (0.917) and RMSD (0.50 Å) for molecular dynamic (MD) simulation.

**Figure 4:**
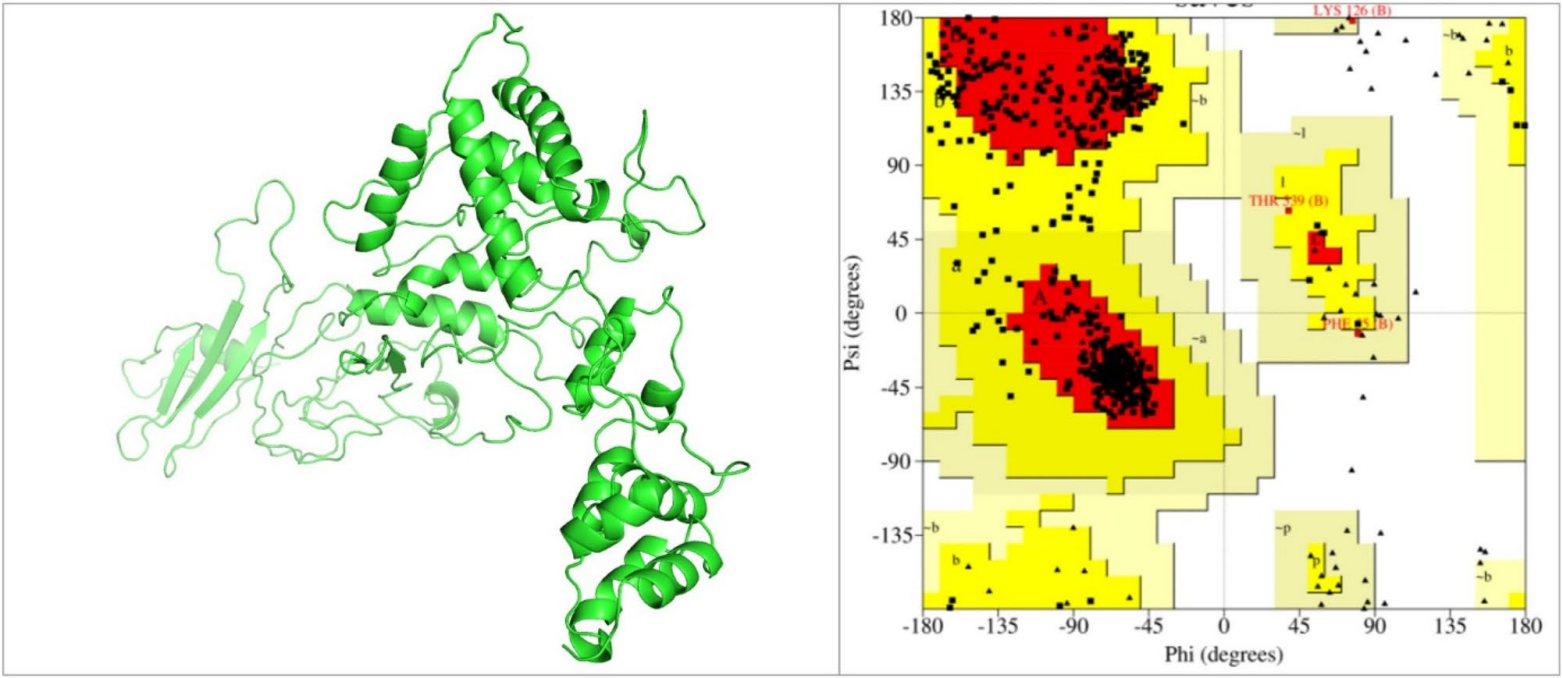
3D structure of final subunit multi-epitopes vaccine construct (left: created using Pymol version 2.3), and Ramachandran plot (right: obtained from SAVES v6.0 https://saves.mbi.ucla.edu/) analysis shows that 82.2% of the residues are in the most favoured regions 17.1% within the allowed regions and no residue in the disallowed regions.

The validation of the 3D structure was done to check for quality and potential errors. Following the MD simulation, the 3D structure analysis by ProSA-web indicates that the structure had a Z-score of − 6.1, and SAVES ERRAT showed the overall quality factor to be 92.6%, and VERIFY 3D reveals that 81.5% of the amino residues have an average score of ≥ 0.2 in the 3D/1D profile. The Ramachandran plot through PROCHECK indicates that 82.2% of the residues are in the most favoured regions, 17.1% are within the additional allowed regions and 0.7% generously allowed regions with no residue in the disallowed regions (Fig. 4).

### Molecular docking of vaccine construct with receptors

Molecular docking (MD) was performed to study the interaction of the final subunit multi-epitopes vaccine construct, with TLRs and major histocompatibility complexes (MHC 1 and MHC II). The HDock server yielded hundreds of model complexes and the best were selected based on their docking score.

The complex of the multi-epitope vaccine (MEV) with TLR2, TLR 3, and TLR 4 had a binding affinity of − 16.8 kcal/mol, − 19.5 kcal/mol, and − 16.6 kcal/mol also with a dissociation constant at 25 °C of 4.7 × 10^−13^ M, 4.7 × 10^−15^ M, and 6.5 × 10^−13^ M, respectively. Also, the complex of MEV with MHC I and MHC II had a binding affinity of − 11.7 kcal/mol and − 8.11 kcal/mol with a dissociation constant at 25 °C of 2.5 × 10^−9^ M and 1.1 × 10^−6^ M.

The interactions between the docked complexes were analysed using the PDBePISA server. It showed 13 hydrogen bonds and two salt brides within MEV–TLR 2 complex, 13 hydrogen bonds interaction within MEV–TLR 3 complex, and eight hydrogen bond interactions between MEV–TLR 4 complex. The MEV–MHC I complex had 10 hydrogen bond interactions and a salt bridge and the MEV–MHC II complex had six hydrogen bond interactions and a salt bridge.

**Figure 5:**
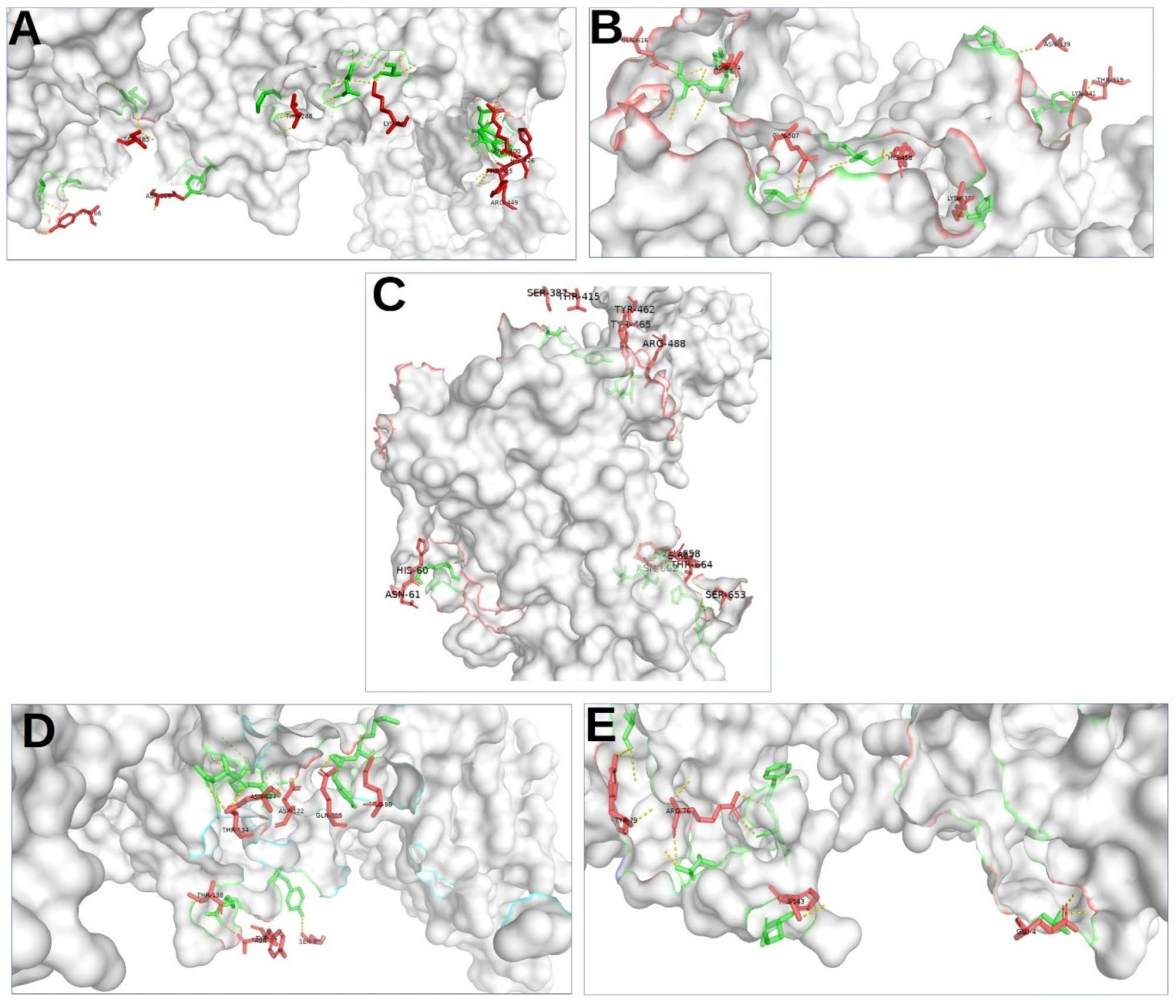
Hydrogen bond interaction map with the pymol of the CCHFV chimeric vaccine in green while the red colour (**A**) TLR-2, (**B**) TLR-3, (**C**) TRR-4MHC-II, (**D**) MHC-I, and (**E**) MHC-II.

The structural evaluation is illustrated in Fig. 5 and the hydrogen bond interactions of the MEV with TLR2, TLR3, and TLR4 are shown in Table 5. The hydrogen bond interactions of the MEV with MHC-I and MHC-II are shown in Table 6.

**Table 5:** Hydrogen bond interactions of the MEV with TLR2, TLR3, and TLR4.

S. no.	TLR2	Å	MEV	TLR3	Å	MEV	TLR4	Å	MEV
1	Arg449	2.69	Ser132	Tyr465	3.58	Leu128	Gln616	3.18	Thr139
2	His426	3.02	Tyr138	Arg488	3.54	Leu128	Gln507	3.55	Ala196
3	Lys347	2.02	Ser200	Arg488	3.31	Ser132	His458	2.40	Ile203
4	Lys347	2.82	Ser200	Ser387	3.04	Ala173	Lys435	3.07	Pro211
5	His318	3.14	Asp204	Thr415	2.86	Ala173	Asn339	3.30	Pro391
6	Lys347	3.29	Asp204	Tyr462	2.54	Tyr174	Lys341	3.74	Tyr395
7	Thr288	3.75	Pro211	Phe657	3.08	Ala206	Thr319	2.47	Tyr395
8	Thr60	3.40	Gly423	Val658	3.88	Thr304	Asn554	3.44	Tyr138
9	Thr65	3.68	Gly428	Asn662	2.30	Leu346			
10	Ser185	2.77	Ile506	Ser653	3.49	Pro389			
11	Phe425	3.82	Tyr135	His60	2.26	Arg285			
12	Arg400	3.80	Tyr138	Asn61	3.30	Arg285			
13	Asp109	2.52	Tyr395	Thr664	3.56	Arg393			

*MEV* multi-epitopes vaccine, *Å* bond distance in angstrom, *TLR* toll-like receptor.

**Table 6:** Hydrogen bond interactions of the MEV with MCH-I and MCH-II.

S. no.	MCH-I	Å	MEV	MCH-II	Å	MEV
1	Thr134	2.61	Asn360	Glu4	3.81	Ala173
2	Asn127	2.85	Asn360	Arg76	3.23	Asp56
3	Ser88	3.46	Tyr395	Arg76	3.47	Phe55
4	Thr134	3.58	Arg361	Tyr79	3.55	Ala36
5	Asp122	3.62	Arg361	His143	3.11	Glu50
6	Gln115	2.68	Phe365	Arg76	2.91	Val46
7	Met98	3.30	Lys366			
8	Tyr84	3.75	Arg393			
9	Asn86	3.23	Arg393			
10	Thr138	3.72	Arg393			

*MEV* multi-epitopes vaccine, *Å* bond distance in angstrom, *MCH* major histocompatibility complex.

### Molecular dynamics simulation

**Figure 6:**
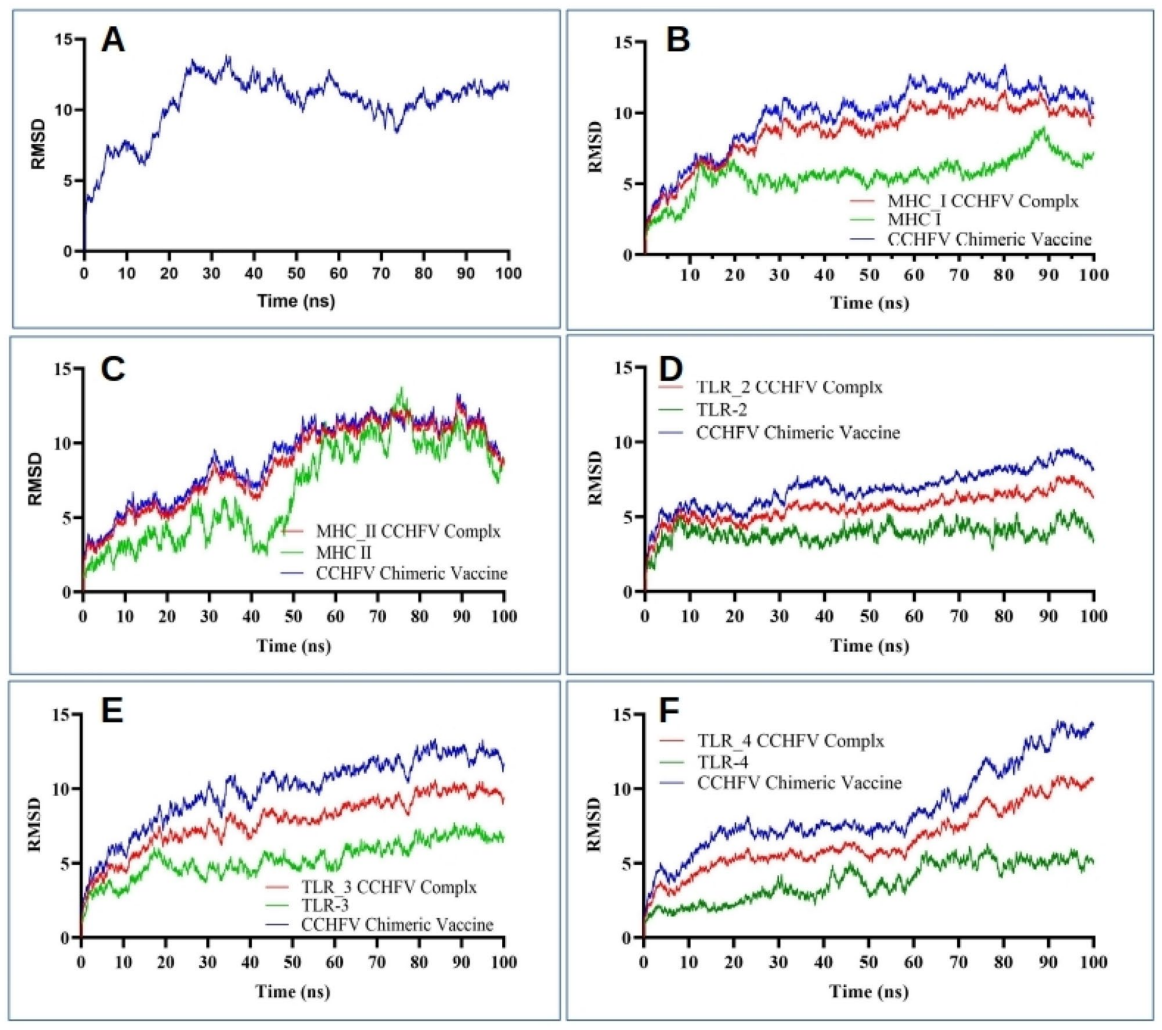
RMSD plot for 100 ns simulation production of (**A**) 3D model CCHFV chimeric vaccine, (**B**) MHC-1 in complex with the chimeric vaccine, (**C**) MHC-II in complex with the chimeric vaccine, (**D**) TLR-2 chimeric vaccine complex, (**E**) TLR-3 chimeric vaccine complex and (**F**) TLR-4 in complex with chimeric vaccine.

The selected complexes were further subjected to MD simulation to evaluate complex stability and residue fluctuation for the period of 100 ns. The temperature, density, and total energy remained stable for the simulation period. The root means square deviation (RMSD) of the complexes appear to converge by the end of simulation production as presented in Fig. 6. Over the simulation period, the average RMSD of MHC-I and MHC-II in complex with CCHFV chimeric vaccine was 8.70 ± 2.08 Å and 8.55 ± 2.79 Å. The average RMSD of TLR-2, TLR-3, and TLR-4 in complex with CCHFV chimeric vaccine was 5.65 ± 0.96 Å, 7.79 ± 1.83 Å and 6.57 ± 2.17 Å (Fig. 6).

The favourable binding free energy as calculated through the MM-GBSA approach reveals that the binding free energy of MHC-I and MHC-II in complex with CCHFV chimeric vaccine − 87.6 ± 11.6 kcal/mol and − 66.7 ± 11.6 kcal/mol. The TLR-2, TLR-3, and TLR-4 in complex with CCHFV chimeric vaccine have the binding free energy of − 82.1 ± 15.93 kcal/mol, − 43.7 ± 8.9 kcal/mol, and − 32.7 ± 15.1 kcal/mol.

### Optimization and in-silico cloning of vaccine construct

The *Drosophila melanogaster-*based expression system was chosen for codon optimization and expression of the vaccine construct because the expression system allows rapid expression and subsequent large-scale, cost-effective transformation and manufacturing of recombinant proteins. The optimization yielded a sequence that is 1701 nucleotides long with a GC-content of 72.4% and CAI of 0.98. The mean GC content of *Drosophila melanogaster* for the adapted vaccine construct sequence was 42.2 suggesting the host is suitable to express the vaccine candidate.

### In silico immune simulation

**Figure 7:**
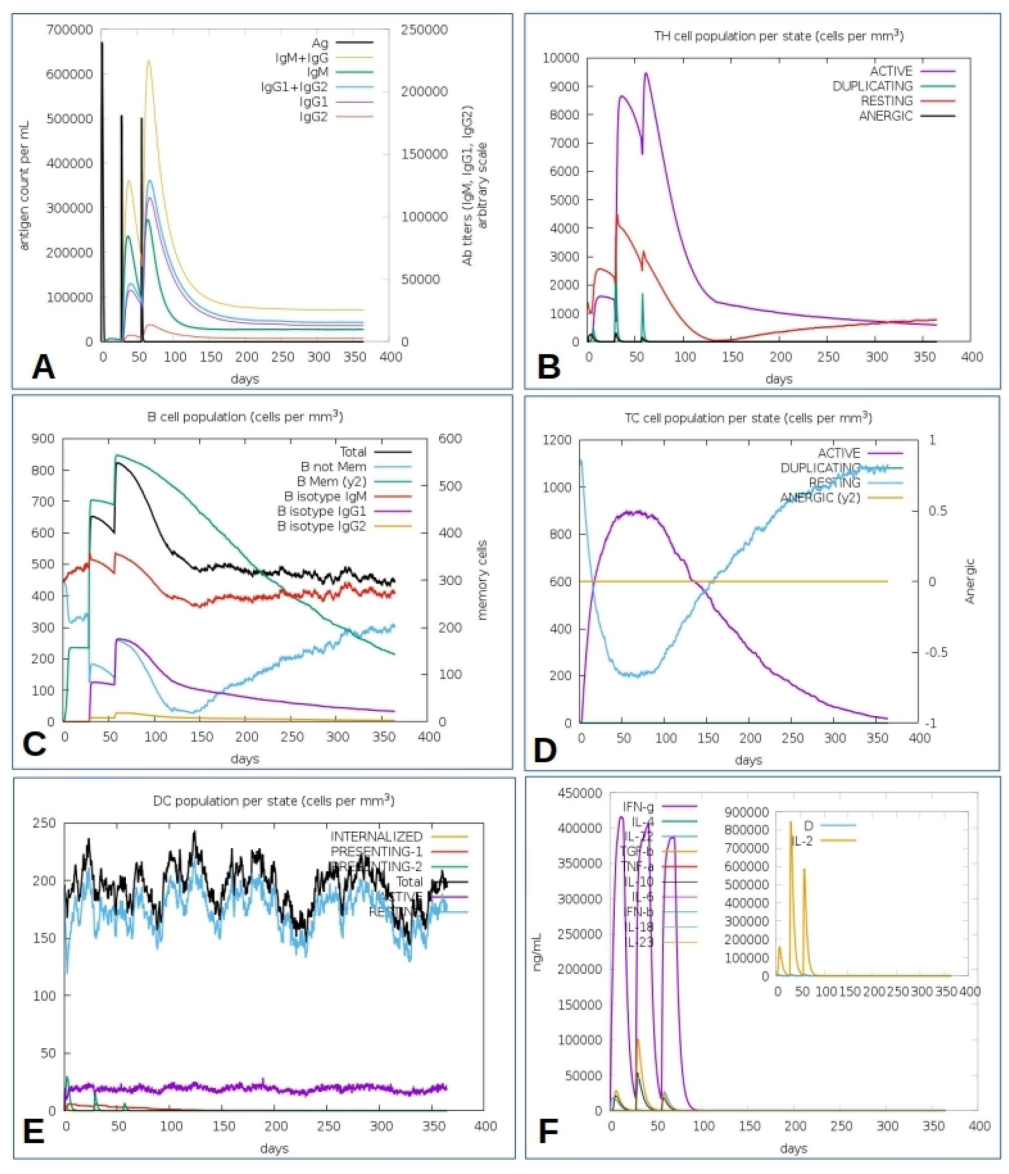
C-ImmSim reveals the immune profile of the CCHFV chimeric vaccine antigen. (**A**) Immunoglobulin production after immunization with sub-type indicated as coloured peaks, (**B**) Evolution of CD4 T-helper lymphocytes count per entity-state, i.e., active, resting, anergic, and duplicating. (**C**) Population of B lymphocytes after three injections with the total count, memory cells, and IgM, IgG1, and IgG2 isotypes, (**D**) CD8 T-cytotoxic lymphocytes count after immunization, (**E**) Dendritic cell population per state which presents antigenic peptides on both MHC class-I and class-II molecules, and (**F**) Concentration of cytokines and interleukins after injection with Simpson D.

The C-ImmuSim server used in simulating the immune profiles of the CCHFV chimeric vaccine revealed that the immune response to the chimeric vaccine was comparable with actual immune responses with higher tertiary and secondary responses. The increased activities of the secondary and tertiary immune responses were noticeable by high levels of IgG1 + IgG2 and IgM and reduced levels of IgG + IgM antibodies (Fig. 7A). The results further reveal the development of immune memory B cells following immunization and increased antigen clearance upon subsequent exposures (Fig. 7B). Consequently, several isotypes of long-lasting B-cell were noticed. This suggests potential switching of the B-cell isotypes and memory formation (Fig. 7C). Similarly, a high response of T-cytotoxic cell populations with respective memory development was observed (Fig. 7D). Following immunization, the continuous proliferation of dendritic cells (Fig. 7E) and elevated levels of IFN-γ and IL-2 with a low Simpson index is apparent (Fig. 7F).

## Discussion

The difficulty in growing multiple organisms, cost of vaccine production, problems with vaccine attenuation, and adverse effects of these vaccines have led to a shift in the development of subunit vaccines using immunoinformatics^91,92^. This approach provides a quick, reliable, cost-effective, and efficient approach to disease vaccine development^93^. The immune-competency of model animals to CCHFV and problems with extensibility and safety issues of available vaccines have delayed production and prevented widespread use of available vaccines, necessitating the development of a new vaccine that may be free of these problems^15,24^. For the development of epitope-based peptide vaccine, the structural proteins are considered as the focus, as they are involved in the interaction between cell receptor and virus particle, its transcription and replication thus playing a significant role in the pathogenesis of the disease^94,95^.

The physicochemical properties of the retrieved proteins were predicted before antigenic determination of their sequences^96^. Using the threshold of 0.4, the viral components were classified into antigens and non-antigens (scores below the threshold) using the VaxiJen server. For accurate component selection, the protegenicity (protective antigen nature), the localization, and transmembrane helices of the protein sequences were further determined^29,97^. This process ascertains the suitability of candidate vaccine selection for experimental validation in the vaccine development process by ensuring that the vaccines do not contain transmembrane helix regions (to ease their expression) and the proteins must not share homology with human proteins to escape the potentiality of causing autoimmune response^98–100^. All the proteins (GPC, NP, and L) have high protegenicity scores and are all localised in the cytoplasm with only NP predicted to have a high adhesive probability among the three proteins with no transmembrane helix. The high adhesive probability predicted for NP would mean it has a high level of adhesion, which has been reported to play a vital role in enabling virus entry and adherence to the host cell^101^. All the predicted proteins were not similar to human, mouse, or pig proteins.

To develop a good vaccine, it must be able to induce immunity through the antigen with a durable adaptive immunity and it should possess antigenic properties, which are important to elicit the immune response of the host^96^.

Using several servers, epitopes for CTL (responsible for developing durable immunity capable of eliminating circulating virus and infected cells) and HTL (associated with the production of both humoral and cellular immune responses by provoking a CD4+ helper T cell response for the generation of protective CD8+ T cell memory and activation of B cells) were selected based on their antigenicity, allergenicity, immunogenicity, and toxicity for the multi-epitope candidate^102,103^. Since IFN-γ secretion is an important mediator of protection against CCHFV, only HTL epitopes that release interferon-gamma (IFN-γ) were chosen for the multi-epitopes construct. To generate the vaccine construct, short peptides (AAY and GPGPG) shown to provoke minimal junctional immunogenicity between the epitopes and a high level of expression and improve the bioactivity of the vaccine were used as linkers between the B and T cells epitopes^104–106^. Using an EAAAK linker, an adjuvant was added to the N terminal of the vaccine as an immunomodulator to develop specific immune responses to antigens, enhancing the stability and longevity of the vaccine against infection^107,108^. In this study, the multi-epitope vaccine construct showed antigenicity with a score of (0.593) as predicted by the VaxiJen server and non-allergenicity with a score of (− 1.03) as predicted by AlgPred. For effective vaccination, a vaccine molecule must provide broad-spectrum protection against different populations around the world. Thus, in designing an epitope-based subunit vaccine, it is important to estimate the fractions of the population in the target endemic zones based on HLA genotypic frequencies. From the results, the selected HTL and CTL epitopes had a widespread coverage of the endemic population of CCHF. The HTL epitopes cover ~ 57% of the Czech Republic, Saudi Arabia, and Poland population, while the CTL epitopes cover ~ 54% of the Czech Republic and Poland population. The multiepitope vaccine construct has a molecular weight of (58.3 kDa), which is within the average molecular weight (40–70 kDa) for a multi-epitope vaccine^28^. The solubility of the vaccine is an important criterion for its creation since the vaccine will be administered in a water milieu in the host body. Subunit vaccines with low solubility have been reported to be disadvantageous in the production of large amounts of virus proteins^109^. Therefore, constructing vaccines with high solubility is a vital requirement for many biochemical and functional analyses^110^. The vaccine construct was predicted to be soluble upon expression signifying easy access to the host. The theoretical pI value of 9.09 and the instability index of 32.1 shows that the vaccine is basic and will remain stable after expression^111^. Based on the GRAVY score and aliphatic index, the result on the hydrophilicity and thermostability indicates that the vaccine construct is hydrophobic making it well-matched for use in endemic areas^112^. The knowledge of the quality of the secondary and tertiary structure of the vaccine construct is of crucial importance in vaccine design (for efficient presentation of antigenic peptides on MHC for triggering strong immune reactions)^105,113^. In this study, the secondary structure analysis showed that the vaccine consisted of alpha helixes, beta-strands, and predominantly coils (44.4%). The tertiary structure was predicted and refined and then the model was assessed in the ProSA web server. The model Z-score was − 6.1, which falls within those commonly observed in similar size-native proteins and the ERRAT overall quality factor was 92.6% revealing that the refined 3D structure of the vaccine is acceptable^28,114,115^. The Ramachandran plot of the refined 3D structure of the vaccine showed that the majority (82.2%) of the residues were in the most-favoured region, with very few residues in the additional allowed regions and no residues in the disallowed regions, demonstrating the excellent quality and stability of the final refined model^115^. The host innate immune responses in vertebrates (retinoic acid-inducible gene I RIG-I)^17^, and Toll-like receptors (TLRs)^18^ have been reported to play a substantial role in limiting CCHFV pathogenesis^22,23^. Also, the role of TLR2 and TLR4 in the recognition of viral structural proteins leading to inflammatory cytokine production has been reported^96,116^. To explore specific interactions and binding affinities of the final subunit multi-epitopes vaccine construct, against TLR2, TLR3, and TLR4 and major histocompatibility complexes MHC I and II, molecular docking were done. The result showed the highest binding affinity between the vaccine construct and TLR3 when compared with TLR2 and TLR4 with numbers of hydrogen bonds. Higher binding affinity was also observed between the vaccine construct and the MHC I complex when compared to MHC II suggesting that the vaccine may have the probability to produce both innate and adaptive immune responses^117^. An MMGBSA analysis revealed that a very small amount of energy is required to bind stable complexes, and MD simulations exhibited very minor fluctuations. Accordingly, these results strongly suggest that the vaccine construct can efficiently bind to the immune receptors.

Expression of the recombinant protein in *Drosophila melanogaster* expression systems is important for validation of vaccine by screening for immunoreactivity through serological analysis^118^. To ensure the complete expression of the designed vaccine protein, codon usage optimization was performed in *Drosophila melanogaster*^119^. The CAI was 0.98 and the GC content was 72.9% in *Drosophila melanogaster*. C-ImmSim simulates the major functional mammal system components bone marrow, thymus, and lymph node^85^. Since a potent vaccine must stimulate an immune response similar to that induced by an antigen with the production of long-lasting adaptive immunity, the response of the immune cells (HTL, CTL, B-cells, dendritic cells, immunoglobulins, and cytokines) was evaluated against the vaccine construct^120^. The immune profiles of the CCHFV chimeric vaccine revealed that the immune response to the chimeric vaccine was comparable with actual immune responses with higher tertiary and secondary responses. Increased activities of the secondary and tertiary immune were noticeable with the production of memory B-cells and T-cells. An increase in levels of IFN-γ and IL-2 following the first injection maintained the peak levels after repeated exposures to the antigen. This indicates high levels of TH cells and thus efficient Ig production, associated with humoral response^121,122^ with a low Simpson index suggesting a possible diverse immune response considering that the constructed chimeric peptide is composed of several B and T epitopes^123^.

## Conclusion

The purpose of this study was to develop a potential vaccine peptide coding for multiple helpers and cytotoxic T-cells that also contain epitopes for B cells. A chimeric vaccine containing these epitopes is likely to have a prophylactic effect, given that the CCHFV proteins contain these epitopes. Based on docking and simulation results, the chimeric vaccine protein had a high affinity and binding potential with immune receptors and remained stable over time. In immune stimulation, models of real-life immune responses were observed. By creating an effective immune memory against CCHFV infections, the chimeric vaccine raised in this study may aid infection control. The next step is to synthesize the peptide in a *Drosophila melanogaster* and proceed with the immunological tests needed to validate the results.

## Supplementary Information

Supplementary Figures.

## Data Availability

All data generated or analysed during the study are included in the submitted manuscript. The sequences of the protein analysed can be retrieved from the GenBank and UniProt database using their ID.
