# Supplementary material for: In silico design and analyses of a multi-epitope vaccine against Crimean-Congo hemorrhagic fever virus through reverse vaccinology and immunoinformatics approaches
Source: Sci Rep. 2022 May 24;12:8736. doi: 10.1038/s41598-022-12651-1 (PMC9127496; doi:10.1038/s41598-022-12651-1)
Supplement: Supplementary file 1 — Supplementary Figures. [file 41598_2022_12651_MOESM1_ESM.docx]

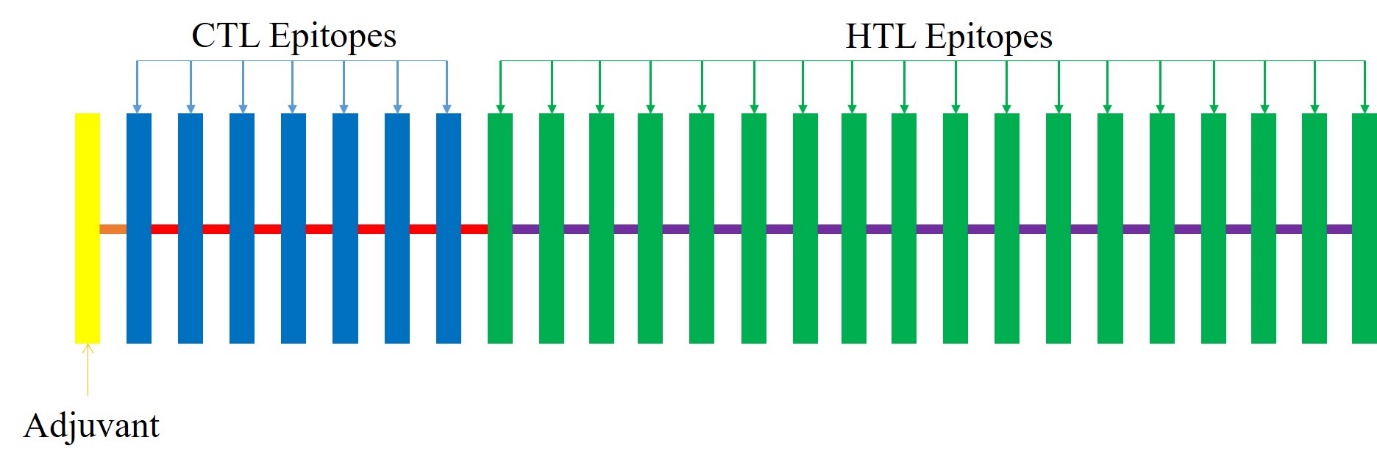


Supplementary figure 1: Construction of the multi-epitope vaccine. Adjuvant (yellow) connected to the first CTL (blue colour) epitope using EAAAK linker(brown colour), the CLT epitopes were joined together by AAY linker (red colour) and HTL epitopes (green colour) joined together using GPGPG linker(Purple).


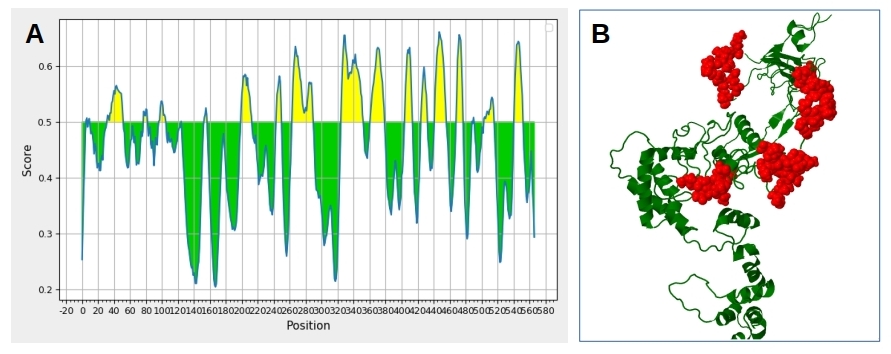


Supplementary figure 2: Predicted linear (A) and conformational B-cell epitopes are portrayed as red spheres (created using Pymol version 2.3) in the chimeric vaccine 3D structure (B)
